# Establishing Ebola Virus Disease (EVD) diagnostics using GeneXpert technology at a mobile laboratory in Liberia: Impact on outbreak response, case management and laboratory systems strengthening

**DOI:** 10.1371/journal.pntd.0006135

**Published:** 2018-01-05

**Authors:** Philomena Raftery, Orla Condell, Christine Wasunna, Jonathan Kpaka, Ruth Zwizwai, Mahmood Nuha, Mosoka Fallah, Maxwell Freeman, Victoria Harris, Mark Miller, April Baller, Moses Massaquoi, Victoria Katawera, John Saindon, Philip Bemah, Esther Hamblion, Evelyn Castle, Desmond Williams, Alex Gasasira, Tolbert Nyenswah

**Affiliations:** 1 EVD Response Team, World Health Organization, Monrovia, Montserrado, Liberia; 2 Laboratory Deptartment, Academic Consortium Combating Ebola in Liberia, Monrovia, Montserrado, Liberia; 3 EVD West Africa Response Team, Foundation for Innovative New Diagnostics, Geneva, Switzerland; 4 Department of Public Health Emergencies, Ministry of Health, Monrovia, Montserrado, Liberia; 5 EVD Response Team, E-health Africa, Monrovia, Montserrado, Liberia; 6 EVD Response Team, United States Centers for Disease Control and Prevention, Monrovia, Montserrado, Liberia; Center for Disease Control and Prevention, UNITED STATES

## Abstract

The 2014–16 Ebola Virus Disease (EVD) outbreak in West Africa highlighted the necessity for readily available, accurate and rapid diagnostics. The magnitude of the outbreak and the re-emergence of clusters of EVD cases following the declaration of interrupted transmission in Liberia, reinforced the need for sustained diagnostics to support surveillance and emergency preparedness. We describe implementation of the Xpert Ebola Assay, a rapid molecular diagnostic test run on the GeneXpert platform, at a mobile laboratory in Liberia and the subsequent impact on EVD outbreak response, case management and laboratory system strengthening. During the period of operation, site coordination, management and operational capacity was supported through a successful collaboration between Ministry of Health (MoH), World Health Organization (WHO) and international partners. A team of Liberian laboratory technicians were trained to conduct EVD diagnostics and the laboratory had capacity to test 64–100 blood specimens per day. Establishment of the laboratory significantly increased the daily testing capacity for EVD in Liberia, from 180 to 250 specimens at a time when the effectiveness of the surveillance system was threatened by insufficient diagnostic capacity. During the 18 months of operation, the laboratory tested a total of 9,063 blood specimens, including 21 EVD positives from six confirmed cases during two outbreaks. Following clearance of the significant backlog of untested EVD specimens in November 2015, a new cluster of EVD cases was detected at the laboratory. Collaboration between surveillance and laboratory coordination teams during this and a later outbreak in March 2016, facilitated timely and targeted response interventions. Specimens taken from cases during both outbreaks were analysed at the laboratory with results informing clinical management of patients and discharge decisions. The GeneXpert platform is easy to use, has relatively low running costs and can be integrated into other national diagnostic algorithms. The technology has on average a 2-hour sample-to-result time and allows for single specimen testing to overcome potential delays of batching. This model of a mobile laboratory equipped with Xpert Ebola test, staffed by local laboratory technicians, could serve to strengthen outbreak preparedness and response for future outbreaks of EVD in Liberia and the region.

## Author summary

We describe a model of a mobile laboratory co-located at an Ebola treatment unit (ETU) equipped with GeneXpert molecular diagnostic platform and automated qRT-PCR test for Ebola virus disease (EVD). The laboratory contributed significantly to the EVD response in Liberia, including supporting surveillance, outbreak detection, contact tracing and management of confirmed cases. The laboratory was run by local laboratory technicians as opposed to international experts on short missions, making it a more sustainable option in prolonged outbreaks and early recovery phases. The redeployment and strategic placement of these GeneXpert instruments to complement EVD isolation facilities throughout the country has strengthened preparedness and response capabilities for future EVD outbreaks in Liberia.

## Introduction

The Ebola Virus Disease (EVD) outbreak first declared in Guinea in March 2014, spread to neighbouring countries Liberia and Sierra Leone and was declared a Public Health Emergency of International Concern (PHEIC) on the 8^th^ of August, 2014 [1]. This deadly outbreak affected some of the least developed countries in the world, with already fragile health systems and insufficient laboratory infrastructure and diagnostic capabilities [2–5]. An unprecedented 28,616 cases and 11,310 deaths were reported across all affected countries, of which nearly 11,000 cases and over 4,800 deaths were reported in Liberia [6]. Failures in diagnostic preparedness, the unavailability of suitable rapid diagnostic tests, the weak surveillance system leading to a delays in detecting cases and the limited therapeutic options for EVD, among other factors, contributed to its rapid spread, and persistent transmission in the affected countries [7–11].

Most commonly, laboratory diagnosis of EVD is achieved by detection of viral RNA, by real-time reverse transcription polymerase chain reaction (RT-PCR) and viral antigen detection tests [7, 12, 13]. Nucleic acid and viral antigen can be detected in blood within 3–10 days after the onset of symptoms [7]. In addition to case confirmation, EVD laboratory data informs contact tracing and emergency response interventions as well as patient management, clinical treatment and discharge decisions [5, 7, 14].

In October 2014, World Health Organization (WHO) issued a target product profile (TPP) for manufactures to develop rapid and easy to use point-of-care diagnostics [15] and an emergency use authorisation (EUA) mechanism was established to review submissions [16]. Foundation for Innovative New Diagnostics (FIND) worked collaboratively with The Emerging and Dangerous Pathogens Laboratory Network (EDPLN), WHO and partners leading field evaluations of novel in-vitro diagnostics [17, 18].

Prior to 2014, laboratory diagnosis for EVD was not available in Liberia and very limited expertise in molecular diagnostic techniques existed, only at the National Reference Laboratory (NRL) in Margibi County. Deployment of international support often as mobile laboratories was critical to the management of the EVD outbreak throughout West Africa [5, 9, 17, 19–21]. At the peak of the outbreak Liberia had ten operational EVD Laboratories supported by international partners [22]. However, as the outbreak waned and specimen numbers declined, international partners exited Liberia leaving only three operational laboratories by June 2015 [22]. These were located at the NRL, Margibi, Phebe Hospital, Bong and Tappita Hospital, Nimba, with no EVD laboratory in the most densely populated Montserrado county where the capital city Monrovia is located.

The magnitude of the outbreak and the re-emergence of clusters of EVD cases following the declaration of interrupted transmission, highlighted the need for sustained EVD diagnostics to support surveillance in Liberia [2, 5, 7]. Following the Margibi cluster in June 2015 [23, 24], Liberia entered a 90 day period of heightened surveillance. Subsequently, an unanticipated increase in specimens taken from patients fitting the EVD outbreak suspect case definition were received at the three operational laboratories between August-October 2015. Insufficient laboratory diagnostic capacity, resulted in an inability to sustain real-time testing and a build-up of untested specimens in the system. A number of measures were imposed to increase daily EVD diagnostic capacity from 180 to approx. 250 specimens including; extended laboratory hours of operation, training local laboratory technicians on molecular diagnostics and the implementation of the Xpert Ebola Assay on the GeneXpert system.

The Xpert Ebola test, is an automated cartridge-based system for both RNA extraction and RT-PCR detection of Ebola virus (EBOV), nucleoprotein (NP) and glycoprotein (GP) gene targets [7, 25–27]. The system is designed for the rapid testing of suspected and confirmed EVD cases in health-care settings such as isolation facilities and Ebola Treatment Units (ETU) [7]. Xpert Ebola Assay is typically used for the detection of EBOV in whole blood samples and has no detectable cross-reactivity with arboviruses or other haemorrhagic fever viruses [26, 28]. The result provides a cycle threshold (Ct) value for the RT-PCR, which has previously been shown to inversely correlate with the quantity of viral target present in the specimen [19, 21, 26]. Low Ct values upon initial testing indicate high viral loads in patient samples and more severe disease with a higher case fatality rate [7, 19, 21, 29–32].

The WHO included Cepheid’s Xpert Ebola Test to its list of Ebola diagnostics with EUA on the 8^th^ May 2015 and it received Food and Drug Association (FDA) approval in March 2015 [33, 34]. In September 2015, the Xpert Ebola Assay was approved by the Liberian Ministry of Health (MoH), for use as a stand-alone diagnostic test for EVD in whole blood specimens. With support from WHO, FIND, Academic Consortium to Combating Ebola in Liberia (ACCEL), United States Centers for Disease Control and Prevention (CDC) and other stakeholders, the Xpert Ebola Assay was instituted at a mobile laboratory, the Eternal Love Winning Africa (ELWA) III Laboratory. We describe the establishment, operations and impact of the ELWA III mobile laboratory over the 18-month period of operation.

## Methods

### Ethics statement

This public health intervention was part of the Ebola outbreak response in Liberia and was a humanitarian project approved by the Ministry of Health (MoH), Liberia and resourced and supported by a collaboration of international partners. Data analysis was performed on existing, anonymized datasets that had been previously analysed and reported through the established MoH laboratory EVD results reporting system. Secondary analysis of pre-existing, anonymized data did not require ethical review and approval by the Liberia National Research Ethics Board (NREB).

### Establishment and coordination of the mobile laboratory

The mobile laboratory structures described here were donated to the MoH following the departure of international partners (Dutch Mobile Lab) in early 2015. In August 2015 WHO and CDC logistics teams facilitated the transfer of the mobile containers from Sinje, Grand Cape Mount county, approximately 32 kilometres / 20 miles to be located at ELWA ETU in Montserrado county, the only remaining functional ETU in Liberia.

**Fig 1:**
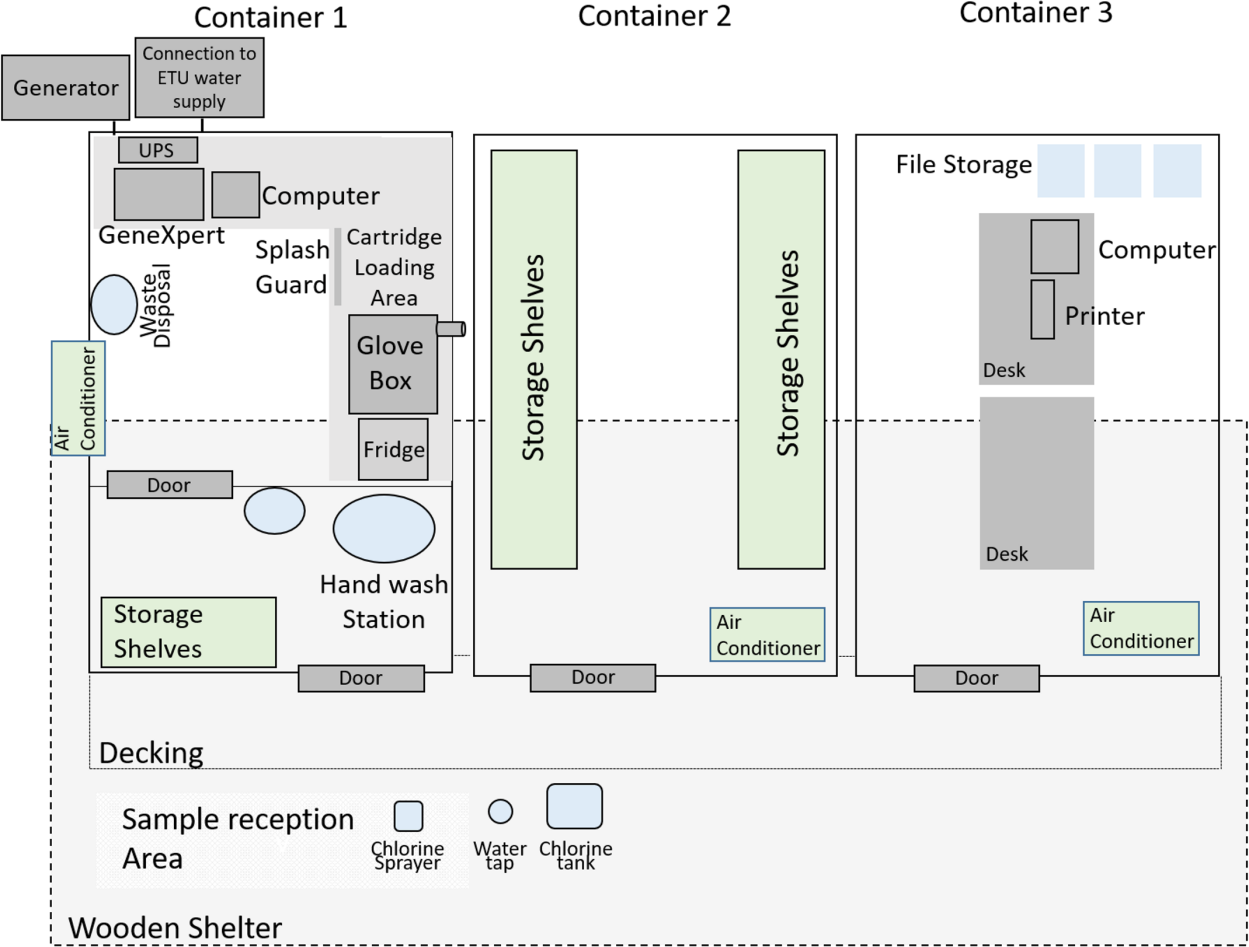
Representation of the layout of the ELWA-III mobile Ebola testing laboratory, at the ELWA Ebola treatment unit, Monrovia.

The mobile unit consisted of three separate containers; the laboratory unit, a storage unit and an office. The layout of the laboratory is represented in Fig 1. A sample reception area was erected in front of the laboratory and roofing was constructed to cover this area and the containers to protect against harsh weather conditions. The laboratory container was pre-fitted with a glove box and filters, ventilation system, an uninterrupted power supply (UPS), a refrigerator and an air-conditioning unit.

### Laboratory coordination

**Fig 2:**
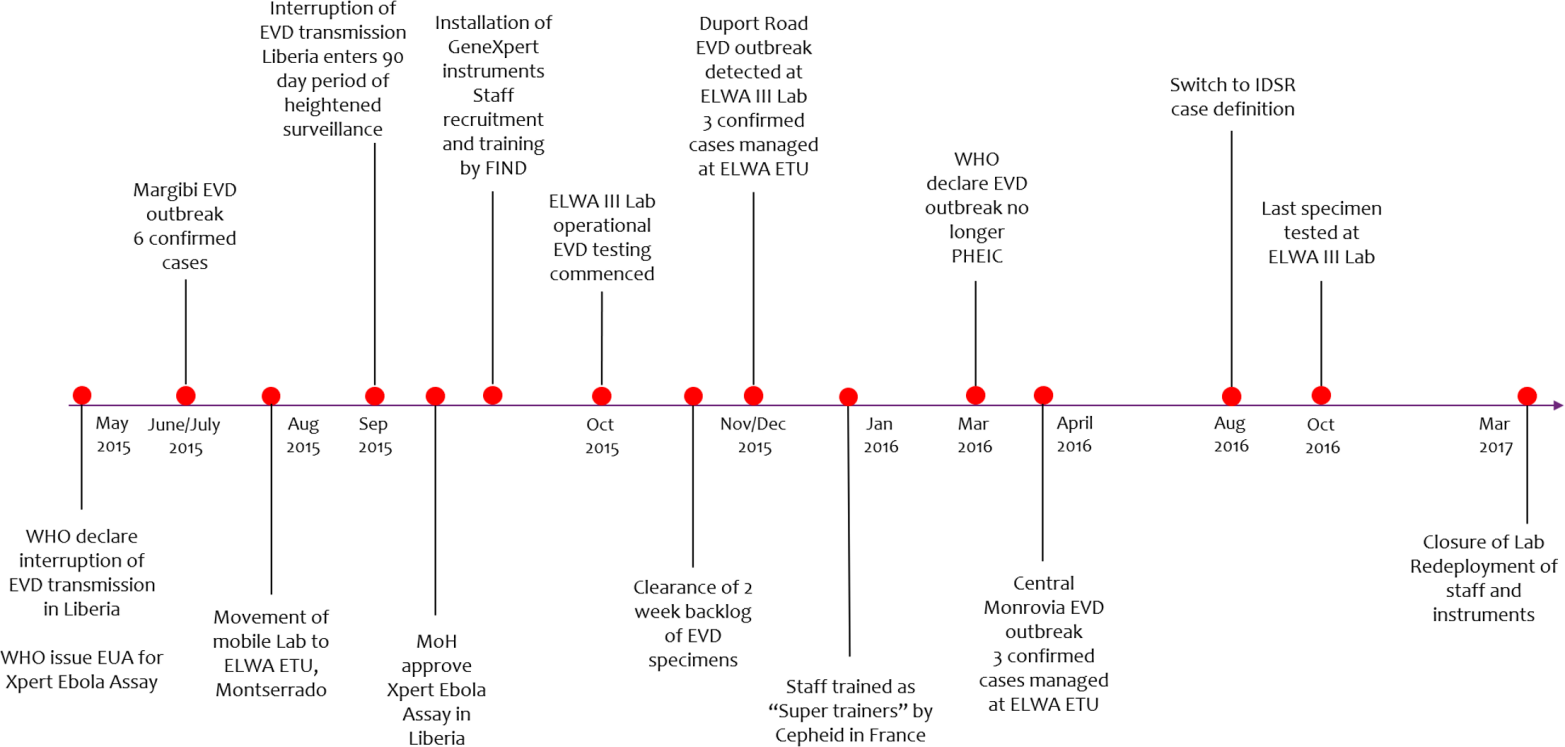
Timeline of key events in establishing and running the ELWA-III mobile EVD testing laboratory—September 2015 to March 2017.

Fig 2 shows the timeline of key events in establishing and running the ELWA III mobile EVD testing laboratory from conceptualisation and operationalisation of the laboratory in 2015 to testing of the last specimens in October 2016 and eventual closure in March 2017. Under the coordination of MoH and WHO, the project was successfully implemented through a collaborative framework involving a number of key laboratory stakeholders; MoH, WHO, ACCEL, FIND, eHealth Africa, Riders for Health (RFH) and US CDC. WHO and ACCEL provided oversight and coordination including technical guidance, logistic support, staff management, training and data management during the 18-month operational period described.

### Operational logistics

The ELWA III laboratory provided diagnostics for specimens collected at the ELWA ETU as well as surveillance specimens collected from Montserrado county and the surrounding areas. In the initial months of operation the laboratory also acted as surge capacity for the other three EVD testing laboratories where the number of specimens being received for testing was higher than the daily testing capacity. Transport of specimens to the ELWA III laboratory was facilitated by RFH, the established specimen transportation system in Liberia. RFH is a is an international non-profit organisation that provides innovative solutions for specimen transport in many African countries from rural health facilities to diagnostics centres using motorcycles [35].

Four GeneXpert instruments (each with a 4-module configuration) were installed within the laboratory container along with a shared laptop, individual power stabilisers and bench shields. Three instruments were donated by FIND and one by ACCEL as part of EVD response efforts in Liberia. The Xpert Ebola Assay kits (50 tests each) were provided by FIND and WHO and stored in the air-conditioned storage unit in accordance with manufacturer’s instructions (2–28°C) [Xpert Ebola IVD package insert 301–4826, revision A, June 2015] with surge capacity stored at WHO warehouse. The cost of the GeneXpert instruments was US$ 17,000 each, while that of a single cartridge during this time was US$ 19.80.

Onsite at the ETU two designated independent generators (10kva Kipor and KOHLER-SDMO TE Silence) provided 24 hour power supply to the laboratory. Fuel was procured by WHO requiring approx. 15 gallons per day. Continuous power maintained the air-conditioning and refrigerator enabling daily operations during working hours and stable storage of reagents and inactivated samples. Running water was provided by connection to the onsite ETU water system. Biohazardous waste was collected in biohazardous waste bags, sealed at the laboratory and brought to the ETU onsite incinerator daily (Alba INCINER8). Due to the automated cartridge based system the only chemical waste generated was disinfectant solution which was disposed through the ETU disposal system. Fresh chlorine solution was obtained daily from the ETU and buckets and a spray can were refilled daily to disinfect all specimen containers and forms before entering the laboratory. Personal protective equipment, biohazard bins, biohazard bags, gloves, face shields, bench shields, cleaning supplies and all additional laboratory supplies were provided by MoH and partners. (S1 Table)

Internet was provided through a mobile 3G modem preloaded with data bundles, sourced from a Liberian telecommunications company and required approximately 10 USD per week for operations. A designated laptop, phone and internet connection facilitated daily reporting of results to the incident management system (IMS) and stakeholders. 24 hour/7 security services were available in the form of a night-watch security guard, employed through the ETU. A designated vehicle and driver were provided by WHO to transport staff between their homes and the laboratory.

### Human resources and training

In September 2015, six Liberian laboratory technicians were recruited and trained on the GeneXpert diagnostic system at ELWA III laboratory by the regional FIND representative (S1 Text). The week-long training covered technical knowledge and practical skills about the GeneXpert system and Xpert Ebola Assay. Pre- and post-testing were conducted as well as written and practical evaluation of testing performance to ensure competence and understanding of the principles of testing, troubleshooting and data management. Bench checklists were provided to ensure that stock checking, laboratory decontamination and temperature recording are done daily. Picture aids helped to ensure that testing protocol was followed. On-site supervisory visits formed a critical part of the quality assurance programme associated with Xpert Ebola implementation, and were conducted at predetermined time intervals by FIND for early identification of any problems related to staff, reagents or instruments. Refresher training was conducted as and when needed. The Laboratory’s Standard Operating Procedures (SOPs) and work flow were jointly developed and implemented by WHO and ACCEL (S2 Text).

WHO and ACCEL trained laboratory staff on specimen reception and biosafety. WHO and eHealth Africa (CDC-funded implementing partners) facilitated data management and reporting of results including a full-time data manager. Training and supportive supervision for data management was provided by WHO onsite over the course of a month, with periodic monitoring site-visits throughout the operation of the laboratory.

In January 2016, two staff members were supported to attend advanced training through Cepheid’s High Burden and Developing Countries (HBDC) Training Programme in Toulouse, France. This specialised training enabled them to serve as “super trainers” responsible for basic equipment maintenance, troubleshooting, calibration and quality assurance of the Xpert Ebola test and providing ongoing training and mentoring for other staff. Basic equipment maintenance was provided by super-trainers as per manufacturers schedule while more complex equipment maintenance and technical support was provided remotely by FIND’s regional representative and Cepheid.

Walk-throughs and mock-tests were completed before testing was initiated in September 2015. For all laboratory operations, the buddy-system was adopted whereby two technicians and the laboratory site manager monitor each other’s safety and remain in constant consultation throughout the process. In the initial weeks of operation, staff worked on a shift basis over six days a week, with four staff covering a 12 hour working day. In line with a decreased workload in November 2015, working days were reduced to eight hours with two technicians working in teams on alternate days. The full-time site manager employed by ACCEL oversaw day-to-day operations. The manager was a local laboratory technician with significant experience working at the regional EVD reference laboratory in Tappita, Nimba, where he had been trained in conventional RT-PCR techniques by international laboratory personnel.

During the initial months, daily technical assistance and coordination was provided by WHO and ACCEL. As laboratory technicians grew in competence and independence the level of support required reduced. Regular monthly staff meetings were held at the laboratory and chaired by WHO. In addition, WHO and ACCEL provided assistance in resolving emerging challenges at the laboratory such as power cuts, addressing equipment errors, sourcing facilities and maintenance technicians and handling specimen rejection, amongst others.

### Xpert Ebola assay performance, biosafety and quality assurance

Specimens were delivered to the laboratory either from the ETU or the catchment area by RFH couriers. All specimens were transported in triple packaging and RFH couriers were trained on biosafety for handling EVD specimens. Upon arrival at the laboratory plastic containers were placed in a custom build wooden box in the specimen reception area and forms received by the laboratory technicians. Containers were disinfected in a bucket of 0.5% hypochlorite for >20mins. Forms were sprayed by a laboratory technician with 0.5% hypochlorite and allowed to air-dry. Disinfected containers were then transferred to the glove box in the laboratory where secondary packaging (plastic bags) was removed. Differential pressure in the glovebox was maintained at 20–50 Pa. Individual specimen tubes were labelled and matched with the corresponding form which was held outside the glovebox, requiring two technicians to assign unique laboratory numbers. Forms were then transferred to the office where the data manager entered all details into the approved MoH EVD laboratory database (Microsoft Access).

The Xpert Ebola assay was carried out following Cepheid’s instructions [Xpert Ebola IVD package insert 301–4826, revision A, June 2015]. Briefly, the whole blood specimens were inactivated within the glove box by adding an aliquot/soaked swab of the blood sample to a pre-labeled vial containing Xpert inactivation buffer. Following a further 20-minute hypochlorite decontamination step, vials of inactivated blood sample were wiped down with 70% ethanol and removed from the glovebox. Processing of the sample from this stage onwards was considered safe on the bench. An aliquot of the inactivated blood sample was then pipetted into the corresponding pre-labelled, room temperature Xpert cartridge and the test started within 30 minutes using Cepheid’s specifications. The remaining inactivated blood samples were stored at 4°C for up to 72 hours in case repeat testing was required. Results were available after 1 hour 30 minutes.

For each test performed, the instrument, system and cartridge contents were monitored and checked for quality, specifically; sample processing, cartridge reagents, instrument check and PCR conditions. Two internal controls are included within each cartridge; the, sample adequacy control (SAC), a human housekeeping gene known as hydroxymethylbilane synthase, confirms that sufficient host cellular material was present and detectable in the sample and the exogenous sample processing control (SPC) controls for PCR inhibition [27].

The probes and primers in this assay detect two structural genes, glycoprotein (GP) and nucleoprotein (NP) of the EBOV genome. Positive tests were those determined as reactive on both GP and NP targets by the GeneXpert software. Negative tests were those determined as non-reactive on both GP and NP targets by the GeneXpert software. A result of invalid was reported when the assay was processed with errors; either SAC failed, CIC failed, or both SAC and CIC failed. A result of indeterminate was reported when the assay was processed without any errors but a specimen could not be determined as positive or negative. This happened when one target NP or GP was detected without the other; NP detected; GP not detected or NP not detected; GP detected.

All positive specimens were retested on the GeneXpert system and also transported to the NRL for confirmation using the US Department of Defence (DOD) EZ1 assay and storage in the -80 freezer on-site. All invalids were repeated and if a second invalid result was obtained the specimen was referred to the NRL for confirmation and/or a repeat specimen requested. Xpert check was carried out weekly to verify optical and thermal functionalities of the instruments.

### Data management and results reporting

Results from the instruments were manually written into a daily worklist corresponding to the unique laboratory number by the laboratory technician which was then used to transfer results to the MS access spreadsheet by the data manager. Priority specimen results were phoned immediately to MoH and WHO Laboratory Coordinators who then informed requesting epidemiologist/clinician and stakeholders.

A data manager entered all patient data into the EVD laboratory database as specimens were received at the laboratory. Results were then matched with the patient information in the database. At the end of each day the patient and laboratory data were exported into the MoH reporting template (Microsoft Excel) double-checked and reported to a MoH-approved list of recipients including IMS members, County health teams (CHT’s) and stakeholders via email. This information was also uploaded into the WHO EDPLN database which produced WHO weekly situation reports on the outbreak [36]. Paper records of laboratory specimens and results, specimen submission forms and laboratory logbooks were stored at the laboratory, accessible only to authorized personnel.

An SOP for handling priority specimens was developed by the inter-organizational laboratory team. During the period of enhanced and thereafter routine EVD surveillance, a specimen was classified as “priority” when obtained from a presumptive/highly suspect EVD case through clinical judgement or epi-linkage. Once identified, priority specimens were transported urgently to the laboratory, triaged and processed immediately and results reported back by phone to the surveillance or case management teams as soon as available.

### Staff feedback

In February 2017, as part of the site close-out activities, a group forum using a short questionnaire was held with the laboratory staff. The intention was to gain an insight into the experience of the Liberian staff and identify best practices and opportunities for integration in future operations of mobile laboratories. Key issues explored included level of staff satisfaction while working at the laboratory, biosafety and security concerns of staff, contribution of the staff to the Liberian EVD response, opportunities presented by the response, challenges experienced in day-to-day work and the significance, if any, of working in a multi-stakeholder collaborative environment.

## Results

### Testing capacity and impact on the surveillance system

**Fig 3:**
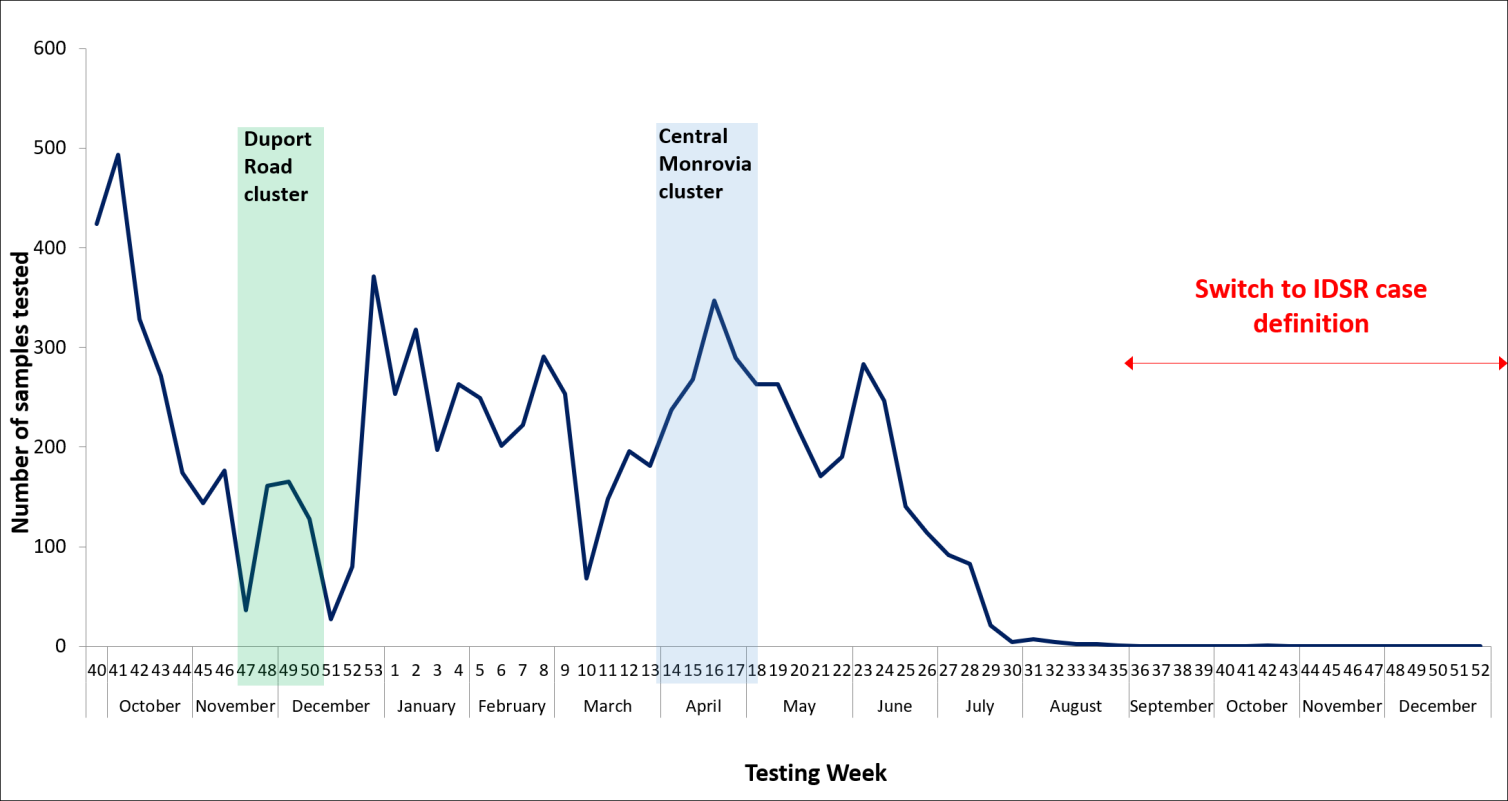
Number of EVD specimens tested at ELWA III laboratory from initiation of testing in September 2015 to the end of 2016; displaying Duport Road and Central Monrovia clusters and the impact of the change to IDSR case definition on the numbers of specimens being processed at the laboratory.

From its establishment on 28 September 2015 to the end of March 2017 the ELWA laboratory tested a total of 9,063 blood specimens, including 21 positive from six confirmed cases, 9,039 negative, two indeterminate and one invalid (Fig 3 and Table 1). Operationalisation of the ELWA laboratory increased the daily testing capacity for EVD in Liberia from 180 to 250 specimens and increased the number of operational EVD laboratories from three to four, providing diagnostics services in Montserrado county.

**Table 1:** No of EVD specimens tested at ELWA III laboratory from September 2015 to the end of 2016, by month of testing and results interpretation.

Month of Testing	Total Samples Tested	Sample Interpretation			
		Negative	Positive	Indeterminate*	Invalid**
**2015**					
Sep	171	171			
Oct	1663	1663			
Nov	623	613	8	1	1
Dec	403	403			
**2016**					
Jan	1149	1149			
Feb	992	992			
Mar	732	732			
Apr	1226	1212	13	1	
May	963	963			
Jun	885	885			
Jul	238	238			
Aug	16	16			
Sep	0				
Oct	1	1			
Nov	0				
Dec	0				
**Total**	**9063**	**9039**	**21**	**2**	**1**

*Indeterminate; NP detected; GP not detected or NP not detected; GP detected

**Invalid; either SAC failed, CIC failed, or both SAC and CIC failed

From September to November 2015, the ELWA laboratory had the capacity to test up to 100 specimens requiring four technicians and 12 hour working days. Following clearance of the specimen backlog, laboratory working hours were reduced and the maximum daily capacity was 64 specimens, requiring two technicians working 8 hours per day. The mean specimens tested per month from September 2015 to August 2016 was 755, ranging from 1663 to 16 (Fig 3). In October 2015, the laboratory processed >1600 specimens to clear the backlog of untested samples in the laboratory system and to re-establish timely diagnostics to support EVD surveillance in Liberia. Following the shift to the more specific Integrated Disease Surveillance and Response (IDSR) case definition (Table 2) in August 2016, the numbers of EVD specimens being taken in the field decreased dramatically (Fig 3). The last specimen was tested at the laboratory in October 2016 and no samples were received in the last five months of operation. The decision to remain operational from January to March 2017 was to support the ETU in the event of a re-emergence or reintroduction of EVD.

**Table 2:** Case definition of suspect case of viral haemorrhagic fever as defined in the Integrated Disease Surveillance and Response (IDSR) Guidelines for Liberia.

	Suspected case
**Outbreak setting**	Any person (alive or dead) with sudden onset of high fever and at least three of the following symptoms: headaches, vomiting, anorexia/loss of appetite, diarrhea, lethargy, stomach pain, aching muscles or joints difficulty swallowing, breath difficulties, hiccups; OR Any person with acute fever and inexplicable bleeding; OR Any sudden, inexplicable death; OR Clinical suspicion of VHF OR A person (alive or dead) suffering or having suffered from a sudden onsite of high fever and having had contact with: a dead or sick animal (for Ebola); a mine (for Marburg)
**Routine setting**	Any person, alive or dead, with onset of fever and no response to treatment for the usual causes of fever in the area AND at least one of the following signs: Bloody diarrhea, bleeding from gums, bleeding into skin (purpura), bleeding into eyes or urine OR clinical suspicion for Ebola or Marburg Virus Disease

### EVD cluster detection and outbreak response

Following clearance of the backlog (>800) of EVD specimens in early November 2015, a new case of EVD was identified at ELWA III Laboratory on the 19^th^ November, in a specimen from a 15-year old boy admitted to the tertiary hospital in Monrovia [37]. The IMS was activated and the outbreak referred to as the Duport Road cluster [37]. The confirmed case and family members were transferred to the ETU with two close contacts testing positive upon initial testing. All three cases were admitted to the confirmed ward with other close contacts admitted to the suspect ward.

In March 2016, the Central Monrovia cluster index case (deceased) was confirmed at the laboratory and prompt identification of an additional case in this cluster was facilitated. Patient 2 (CM2) was admitted to the ETU confirmed ward and a number of close contacts admitted to the suspect ward. Patient 3 (CM3) of the Central Monrovia cluster had been admitted to the suspect ward for monitoring and had had one negative test when a raise in temperature prompted re-testing and revealed that the patient had EVD viral RNA in their blood. The patient was immediately transferred to the confirmed ward to avoid potential transmission to other patients being held in the suspect ward.

During these periods, several priority specimens were taken in the field or at primary health care facilities in Montserrado around the response zone. Sample turn-around-time for priority specimens, from receipt at the laboratory to reporting of results was approx. 2–3 hours. The system allows for single specimen testing enabling immediate testing of priority specimens without the need to batch. Line-lists of all confirmed and suspect cases were updated with laboratory results each day, and shared with surveillance and case management teams for discussion at the daily county IMS meeting to ensure implementation of timely and effective response interventions.

### Case management of confirmed cases

**Fig 4:**
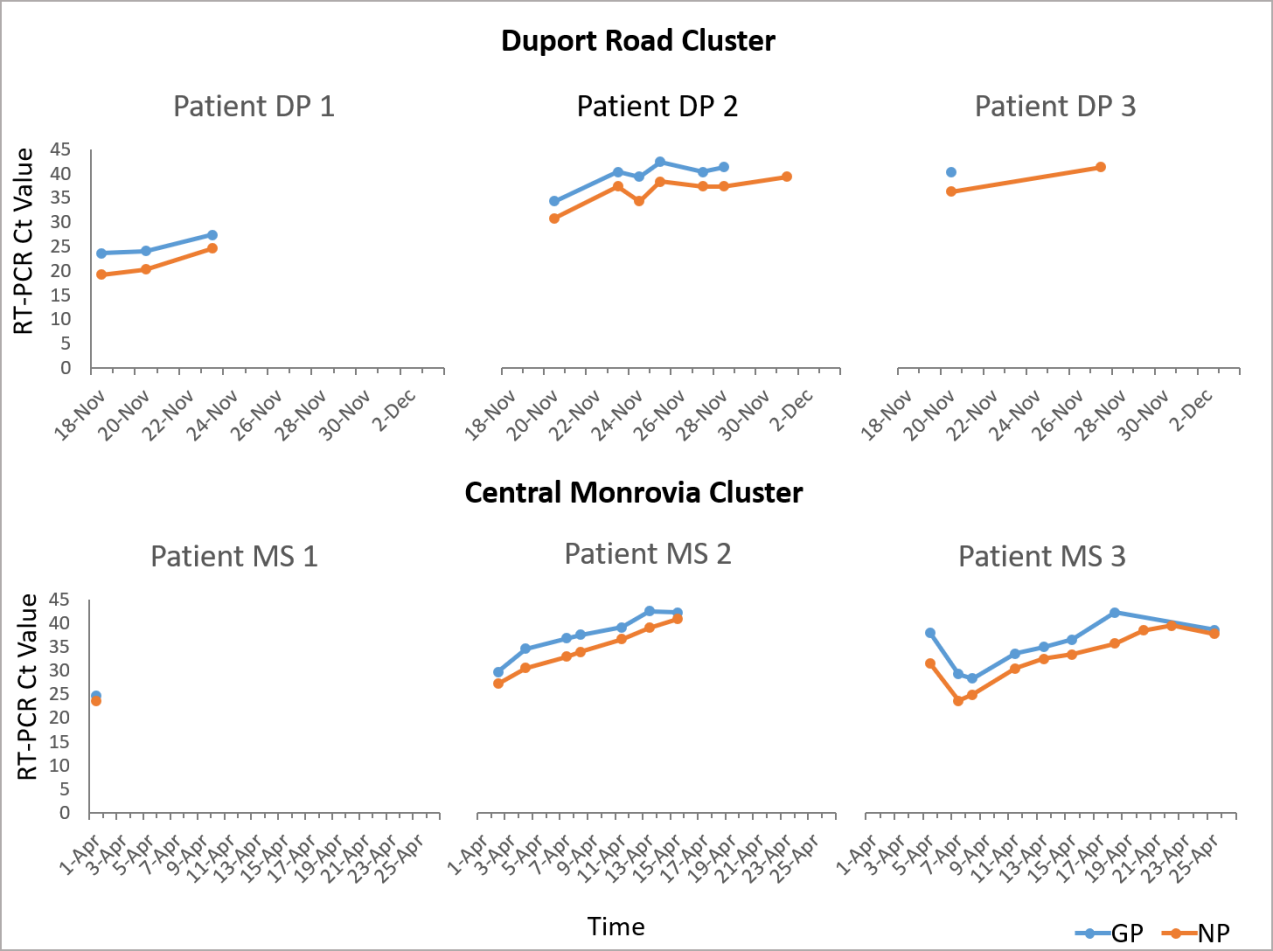
RT-PCR Ct values for NP and GP gene targets from positive blood samples of EVD cases from the Duport Road cluster, November-December 2015 and the Central Monrovia cluster, March-April 2016.

During both the Duport Road cluster (Nov/Dec 2015) (3 confirmed cases) and the Central Monrovia cluster (March/April 2016) (3 confirmed cases), EVD positive patients were monitored regularly by testing repeat specimens at the mobile laboratory. Ct values of consecutive specimens from EVD positive patients were tracked and compared to indicate trends in the viral load of patient specimens (Fig 4). This information was used by the case management team to inform clinical management and discharge decisions. The index cases in both outbreaks, Patient 1 in the Duport Road cluster (DP 1) and Patient 1 in the Central Monrovia cluster (MS 1), had very low Ct values, (<25) upon initial testing indicating high viral loads. Both patients died. Patients 2 and 3 in both clusters (DP 2, 3 and MS 2,3), had higher Ct values upon initial testing, (>25) and these continued to rise gradually over the course of 2–3 weeks until eventual recover and discharge. In specimens taken from patient DP 2 and DP 3, the NP target was detected for longer than the GP target causing results to be reported as indeterminate by the GeneXpert software. Patients were discharged from the ETU once two consecutive specimens taken 48 hours apart tested negative for both GP and NP targets.

### Findings of staff feedback forum

All four of the laboratory technicians reported the overall experience of working at ELWA III laboratory as very rewarding and they felt very proud of their contribution to the EVD response in Liberia. All staff members believed that the coordination and collaborative efforts of partners and stakeholders required to run the laboratory was successful and resulted in very positive outcomes. Staff described the laboratory environment and the work as both challenging and rewarding and that the skills and experience gained were beneficial for their future careers. Overall staff felt valued and supported in their work at the laboratory and benefited from additional training opportunities which would otherwise not have been available. They regarded their greatest achievements as identifying the last clusters of EVD cases and helping to end the outbreak in Liberia. The greatest challenges reported by staff were dealing with the specimen backlog upon opening of the laboratory and adapting to working in the confined space of a mobile laboratory.

### Resolving challenges at the mobile facility

The main challenge at the laboratory was the huge workload in the initial months of operation which required staff to work 12-hour days. In the first weeks the glove box sustained rain damage and was not operational while HEPA filters had to be sourced internationally. This situation was overcome by processing specimens at the NRL then transferring inactivated samples to ELWA III for processing on the GeneXperts each day, a process requiring a high level of coordination and support from staff at the NRL. There was no onsite storage capability for “live” blood samples; these had to be inactivated upon receipt and stored overnight for next day processing. Specimens were stable in inactivation buffer refrigerated for >24 hours according to Cepheid instructions.

As previously reported during the outbreak, biosafety of specimen collection and transport was not optimal and specimens were often rejected due to inappropriate or unsafe packaging [20]. Laboratory requisition forms were frequently incorrectly filled in, as a result it was often challenging to match a laboratory result to the correct patient, many laboratory tests were reported without a name or identifier, although all were negative. This improved as a unique patient identifier system was implemented in Liberia, but never fully resolved. To address these challenges, safe blood specimen collection and packaging training was conducted in the response zones during both outbreaks and WHO provided blood collection tubes and packaging countrywide throughout the EVD response. County diagnostic officers supported by WHO field staff in each county were trained and responsible for distribution of supplies and quality assurance of specimen collection in their counties. RFH couriers underwent regular refresher training on good biosafety practices.

Results dissemination of surveillance specimens from central to county and facility level was an ongoing challenge throughout Liberia. This was addressed by daily reporting directly from laboratories to CHT’s who were then held accountable for relaying the results to the respective health facilities at the district level.

Following the switch to IDSR case definition in September 2016 when specimen numbers declined drastically, a transition plan for ELWA III laboratory was drafted and agreed by MoH and partners. This outlined redeployment of the instruments, materials and staff from the ELWA Laboratory to support EVD diagnostics at isolation units in designated health facilities around the country. Instruments were redeployed to Tellewoyan Hospital, Lofa; Liberian Government Hospital-Tubmanburg, Bomi; ELWA Hospital, Montserrado; Redemption Hospital, Monrovia. Laboratory technicians were reassigned to facilities around Montserrado and Margibi counties to support EVD, TB and HIV testing. At the end of 2016 hundreds of cartridges supplied by FIND based on testing projections, expired and could not be used for EVD testing.

## Discussion

WHO, Geneva, initially declared Liberia free of EVD transmission in May 2015 and the country commenced a 90-day period of heightened surveillance [38]. By this time many international partners supporting the response had scaled down operations or exited Liberia and EVD diagnostic laboratories had reduced from 10 during the height of the epidemic, to three in June 2015 [22]. Following the Margibi outbreak in June, application of the outbreak EVD case definition and recommendations for heightened surveillance resulted in a surge in the number of EVD specimens being taken throughout the country. Coupled with the reduction in laboratory capacity, this led to a build-up of EVD specimens at the laboratories, culminating in significant delays in testing (up to 2 weeks). The consequent lack of visibility in the EVD surveillance system caused significant concern within IMS and was addressed by a number of measures coordinated by the laboratory team, including establishment of a mobile laboratory using GeneXpert technology at ELWA ETU.

This unique model describes a mobile laboratory structure co-located at an ETU which differed from other laboratories deployed during the response in a number of ways [9, 19–21, 39]. The laboratory was equipped with a rapid molecular diagnostic test, Xpert Ebola, allowing single specimen testing, staffed by local laboratory technicians as opposed to international teams on short deployments, supported by MoH and a collaboration of international partners, and provided EVD testing for both surveillance and ETU admissions towards the end of the outbreak when sustained diagnostic to detect new clusters was essential.

Soon after WHO declared Liberia free of active EVD transmission for the second time on 3^rd^ September 2016 [40], as support from international partners was waning, ELWA III mobile laboratory was operationalised. The backlog of unanalysed specimens was cleared and real-time testing capacity restored by the beginning of November 2015. Shortly thereafter, on 19^th^ November 2015, a new case of EVD was detected at the ELWA III laboratory [37, 41]. On 29^th^ March 2016 WHO declared the Ebola PHEIC over, but emphasised the importance of maintaining the capacity and readiness to prevent, detect and respond to any new cases or clusters. The next day saw confirmation of a new EVD case in Liberia and the Central Monrovia cluster [42]. During both of these outbreaks the contribution of ELWA laboratory demonstrated the importance of diagnostic preparedness and the public health impact of rapid diagnostics [7, 9, 43]. This contribution included detection of re-emergence of EVD cases following the declaration of the end of transmission, rapid testing of suspect cases supporting contact tracing and surveillance activities as well as monitoring confirmed cases admitted to the ETU.

Constant communication and timely sharing of information between the surveillance teams operating in the response zones and the laboratory coordination team facilitated efficient testing of suspect cases supporting contact tracing during both responses [14, 37]. Results of priority specimens requested on suspect cases could be reported within 2–3 hours enabling implementation of effective response measures [14, 37]. On many occasions, short turn-around-time to results prevented the need to transfer suspect cases to the ETU allowing patients to remain in the community or health care facility until a negative result was received [14, 37]. This reduced disruption to lives of the patients and subsequent costs for transport and ETU admission [14]. Fallah et al. described how field blood draw combined with Xpert Ebola diagnostics has the potential to accelerate diagnosis in the emergency setting thereby reducing transmission and improving chances of recovery [14].

During the Duport Road outbreak, three EVD cases were admitted to ELWA ETU [37] and in April 2016, the Central Monrovia cluster, two cases were admitted to the confirmed ward (index case deceased). Co-location of the laboratory onsite at the ETU facilitated rapid testing of cases which allowed for close monitoring of Ct values, thus providing information on patient viral load and stage of disease of each patient, important information for contact tracing and case management teams. Interestingly, in both outbreaks the index case had a very low Ct value (<25) indicating a high viral load at the time of initial testing and neither patient survived. This phenomenon has been reported previously with low Ct value indicating a high viral load at the time of detection and possibly more severe or progressed disease with a higher case fatality rate [19, 21, 29–32, 44]. The collaboration of partners working together under the coordination of MoH and WHO, enabled a high level of situational awareness and supported more timely and informed decision-making in particular where additional tests were required, such as genetic sequencing and serological analysis [14, 37]. In addition, the laboratory informed patient discharge as well as guiding effective treatment interventions.

As described by Semper, the NP target is often detected for longer than the GP target and in higher quantities [27]. In our experience, the GeneXpert test called these results indeterminate (GP not detected, NP detected) and upon retesting at the NRL using the DOD EZ1 RT-PCR assay were reported negative, indicating the higher sensitivity of the Xpert Ebola assay. Indeterminate results were obtained in specimens from confirmed cases who were in the recovery phase, with viral loads having dropped to almost undetectable levels.

The Xpert assay met a number of the key features outlined by the WHO in their TPP [15, 16, 27, 34, 45] and in our experience confirmed its potential to transform EVD surveillance and response by shortening the pathway from ordering an EVD test to final diagnosis. The assay has been shown to have a highly accurate performance and is comparable to other commercial assays for EVD [26, 27, 46]. Running on an automated cartridge-based platform means the assay has many benefits over conventional PCR platforms including reduced technical expertise and training requirements, minimal biosafety constraints outside of the glovebox, less sample processing and a faster turn-around time to results [5, 26, 27, 39]. In Liberia, the assay resulted in shorter testing time than other EVD testing methodologies including the DOD EZ1 RT-PCR assay and US CDC’s Ebola virus NP/VP40 RT-PCR assays which were used in the other three Liberian EVD testing laboratories and averaged at 5–6 hours/run. The laboratory was operational 6/7 days per week and for 12 hours per day (8am-8pm) for the initial months. Specimens did not require batching and the system allowed single specimen testing upon receipt at the laboratory. This differed to conventional RT-PCR formats requiring specimen batching meaning results were often not available until the following day. This was incredibly beneficial during the EVD outbreak periods and throughout heightened surveillance for testing high priority specimens taken in the field. Co-location with the ETU further facilitated short turn-around times for confirmed and suspect cases admitted to the ETU excluding the need for specimen transport previously described as a bottleneck to rapid diagnosis [5]. In Liberia the NRL is located in Margibi county, > 1 hour away from central Monrovia and on poorly maintained roads highly susceptible to delays for staff and specimen transport. The decision to locate the ELWA III laboratory at the ETU in Montserrado was advantageous as it provided EVD diagnostics for the Montserrado catchment area including the main tertiary hospital in Monrovia and numerous health facilities in the area.

The Xpert Ebola cartridge contains all reagents necessary to conduct the assay including extraction and amplification, reducing the complexity of the laboratory procedure and allowing for a minimal laboratory infrastructure with sustainable biosafety levels. Allied to this fact, the laboratory workflow for the Xpert Assay is simpler than conventional RT-PCR approaches and therefore requires minimal training for laboratory technicians with training in Liberia taking less than one week, compared with up to three months for conventional RT-PCR methods. In addition, the cost of the cartridge was US$ 19.80 and cheaper compared to other tests used in Liberia; ~US$ 21 for DOD EZ1 assay, US$ 185 for the BioFire’s FilmArray cassette and ~US$ 20 for US CDC Assay. The closed cartridge system of the Xpert Ebola assay means that all materials for testing are contained within the cartridge thereby simplifying the procurement process. The stability of the cartridges at room temperature (2–28°C) allowed for easy delivery to the site of use and storage onsite in an air-conditioned container without any cold chain requirements.

The model described here, differs from many mobile laboratories deployed during the outbreak as it was staffed by local laboratory technicians as opposed to international experts on short missions, providing more sustainable diagnostic capacity, vital as international partner support declined towards the end of the outbreak [5, 9, 19, 39, 47]. Staff feedback further supports the success of the laboratory and the impact of the initiative on laboratory systems strengthening. The level of satisfaction and contribution was very high amongst staff, reporting a very positive experience. Working at the lab helped them to develop skills and opportunities to contribute to their future careers as laboratory technicians. They reported feeling valued by their work supporting the EVD response and believed that they made a significant contribution to their country. Building such expertise at a local level makes a sustainable contribution to the health workforce in Liberia.

Despite these significant advantages, the Xpert system nonetheless relies on specialized equipment, which requires ongoing maintenance and quality assurance. To address this issue in Liberia, a cohort of six laboratory technicians were supported to attend an advanced training course at Cepheid, Toulouse in January 2016, including two who worked at ELWA III laboratory. These staff now provide technical support, training and mentoring and routine monitoring, maintenance and calibration at all GeneXpert sites in the country.

The GeneXpert instrument itself has a small footprint, a further advantage for a mobile laboratory design. While the cost (US$ 17,000) would be prohibitive without donor support the platforms can be integrated to diagnostic algorithms for a number of diseases of public health importance, therefore building laboratory capacity.

We agree with Goodfellow et al., that equipment left unused by the local laboratories due to a lack of training or unsustainability of reagent costs once international teams leave the country should be avoided [8]. To maximise sustainability of the GeneXpert system in Liberia, an implementation plan was developed for roll-out of GeneXpert throughout the country to provide integrated testing for EVD, Human Immunodeficiency virus (HIV) and Tuberculosis (TB). A Transition Plan for ELWA III Laboratory was developed and implemented outlining the decommissioning of the laboratory, but recommending that it remain in stand-by mode in the case of a re-emergence of EVD. The GeneXpert instruments and laboratory staff have been redeployed to other facilities to support TB and HIV testing to ensure optimum use, impact and sustainability of the platforms. The strategic placement of the GeneXpert instruments aims to complement the establishment of EVD isolation facilities to provide on-site diagnostics for EVD and also to support TB and HIV clinics. In addition, the potential to connect all instruments throughout the country to a centrally monitored server add to the attractiveness from the surveillance perspective.

The establishment of an integrated network of GeneXpert laboratories strengthens epidemic preparedness and response capabilities for potential future EVD outbreaks in Liberia. Laboratory technicians trained as “super-trainers” coordinated and carried out instrument installation and trainings at additional sites for EVD, HIV and TB testing. ACCEL has procured a limited number of Ebola Assay kits (~50) to support continuous testing for EVD, but plans are under way to turn this responsibility over to MoH while cartridges for HIV and TB testing are supported through Global Fund. FIND and Global Fund support sustainable GeneXpert diagnostics for TB and HIV in the region including Liberia.

There were however a number of limitations associated with the GeneXpert laboratory model. Firstly, the assay had not been validated for use with body fluids other than whole blood which was a limitation in Liberia where a significant proportion of samples taken in the field were oral swabs from dead bodies. Guidelines for testing additional specimen types should be developed and field tested [7]. Secondly, as outlined by WHO in the TPP for safe, rapid, and cost-effective EVD diagnostic tests and discussed by Broadhurst et al, desired characteristics include minimal power requirements and maintenance needs [7, 15]. The GeneXpert system relies on specialised equipment, a computer, air conditioning and operational glovebox for specimen processing, therefore requires access to uninterrupted power as well as expertise in instrument maintenance, calibration and repair. Thirdly, specimens could not be stored at the laboratory and all positive specimens were transferred to the NRL for storage while negatives were discarded.

The shelf-life of the cartridges when testing commenced in Liberia was only three months however this was extended by Cepheid upon request after additional quality control data became available. Cepheid have committed to maintaining a stock of Xpert Ebola reagents, though new test lots in future outbreaks may have similar issues. In addition, the unanticipated drop in specimen numbers correlating with the change to the IDSR case definition in August 2016 resulted in hundreds of unused cartridges expiring in late 2016. Improved management of stock and supplies is an important learning experience from this project. EQA panels were not available for proficiency testing during the outbreak and development of low cost panels should be considered for future epidemics.

### Conclusion

EVD remains a threat in West Africa and this may not be the last epidemic in this area [8]. The GeneXpert system requires minimal training, limited laboratory infrastructure, procurement of only reagent kits, no cold-storage and can run on a generator. The training of local Liberian staff on the platform has contributed towards strengthening the laboratory system in Liberia for EVD and other disease of public health importance. The combination of GeneXpert technology and the mobile laboratory unit allows for the rapid deployment of diagnostics within ETU’s or isolation facilities, a previously reported bottleneck during the early stages of the outbreak in 2014 [43]. This co-location significantly improves the turnaround time from specimen collection to reporting of results and thereby triggers timely ETU admission of confirmed cases and implementation of response interventions. The short turnaround time supports efficient contact tracing by rapid testing of priority specimens and informs case management through real-time monitoring of patient viral load to inform clinical management and discharge decisions [5, 19, 21, 30, 32]. We propose that the model described here serves to strengthen outbreak preparedness and response for future outbreaks of EVD in Liberia and the region.

## Supporting information

S1 Table List of laboratory supplies. (DOCX)

S1 Text List of training materials and presentations as per Cepheid training package. (DOCX)

S2 Text List of laboratory Standard Operating Procedure’s (SOP’s). (DOCX)
