# Supplementary material for: Establishing Ebola Virus Disease (EVD) diagnostics using GeneXpert technology at a mobile laboratory in Liberia: Impact on outbreak response, case management and laboratory systems strengthening
Source: PLoS Negl Trop Dis. 2018 Jan 5;12(1):e0006135. doi: 10.1371/journal.pntd.0006135 (PMC5755746; doi:10.1371/journal.pntd.0006135)
Supplement: S1 Table — (DOCX) [file pntd.0006135.s001.docx]

**S1 Table: List of Laboratory Supplies**

| ELWA III EVD LAB - INVENTORY SHEET | | |
| --- | --- | --- |
| ITEM | UNIT | QNTY ON HAND |
| GeneXpert kit (buffer, cartridge & pipette) | Box (50) |  |
| GeneXpert Swab | Box (50) |  |
| GeneXpert Spare Module | Set |  |
| Examination glove (small) | Box |  |
| Examination glove (medium) | Box |  |
| Examination glove (large) | Box |  |
| Examination glove (XL) | Box |  |
| Surgical gown (medium) | Pcs |  |
| Surgical gown (XL) | Pcs |  |
| N95 Respirator | Pcs |  |
| Surgical Mask | Pcs |  |
| Goggle | Pcs |  |
| PPE Suit Hooded Coverall (medium) | Pcs |  |
| PPE Suit Hooded Coverall (large) | Pcs |  |
| PPE Suit Hooded Coverall (XL) | Pcs |  |
| PPE Suit Hooded Coverall (XXL) | Pcs |  |
| Face Shield | Pcs |  |
| Biohazard bags (19"x23") | Cs of 200 |  |
| Ethanol, absolute | 2.5 L |  |
| Micro-Chem Plus | Gallon |  |
| Hand sanitizer | Pcs |  |
| Heavy Duty Sprayer | Pcs |  |
| Test Tube | set (100) |  |
| Pippet Stand | Pcs |  |
| Pippet | Pcs |  |
| Water Bottle | Pcs |  |
| Disposable forceps | Pcs |  |
| Desktop and Peripherals Locking Kit | Pcs |  |
| Power cable | pcs |  |
| USB 3-Button Optical Mouse | Pcs |  |
| Cepheid Mouse Pad | Pcs |  |
| Barcode Scanner | Pcs |  |
| Chlorine (Calcium Hypochlorite, hydrated mixture) | Bucket (10kg) |  |
| Roxy Liquid Soap | Pcs |  |
|  |  |  |
