# Supplementary material for: Establishing Ebola Virus Disease (EVD) diagnostics using GeneXpert technology at a mobile laboratory in Liberia: Impact on outbreak response, case management and laboratory systems strengthening
Source: PLoS Negl Trop Dis. 2018 Jan 5;12(1):e0006135. doi: 10.1371/journal.pntd.0006135 (PMC5755746; doi:10.1371/journal.pntd.0006135)
Supplement: S1 Text — (DOCX) [file pntd.0006135.s002.docx]

**S1 Text: List of Training Materials and Presentations as per Cepheid Training Package**

- GeneXpert technology & installation
- Controls (Internal & External Controls)
- Process of the test- Xpert MTB/RIF
- Process of the test- Xpert HIV VL & EID
- Process of the test- Xpert Ebola
- Xpert assays result interpretation
- Maintenance of the System
- Verification & validation of cartridge processing/ software use
- Trouble shooting
- Practical use of tests
