# Supplementary material for: Establishing Ebola Virus Disease (EVD) diagnostics using GeneXpert technology at a mobile laboratory in Liberia: Impact on outbreak response, case management and laboratory systems strengthening
Source: PLoS Negl Trop Dis. 2018 Jan 5;12(1):e0006135. doi: 10.1371/journal.pntd.0006135 (PMC5755746; doi:10.1371/journal.pntd.0006135)
Supplement: S2 Text — (DOCX) [file pntd.0006135.s003.docx]

**S2 Text: List of Laboratory Standard Operating Procedure’s (SOP’s)**

- ELWA III Laboratory SOP
- SOP for handling priority specimens
- SOP for Riders for Health
- SOP for Safe collection of Blood Specimens
- SOP for safe packing of EVD specimens
- SOP for data management and results reporting at ELWA III Laboratory
